# Clinicopathological characteristics and prognosis of primary appendiceal stromal tumors

**DOI:** 10.1186/s12957-018-1524-1

**Published:** 2018-11-16

**Authors:** Bao Zhang, Guo Liang Zheng, Hai Tao Zhu, Yan Zhao, Zhi Chao Zheng

**Affiliations:** 1 China Medical University, No.77 Puhe Road, Shenbei New District, Shenyang, 110013 Liaoning Province People’s Republic of China; 2 Department of Gastric Surgery, Cancer Hospital of China Medical University, Liaoning Cancer Hospital & Institute, No.44 Xiaoheyan Road, Dadong District, Shenyang, 110042 Liaoning Province People’s Republic of China

**Keywords:** Clinicopathological, Characteristics, Prognosis, PASTs, GISTs

## Abstract

**Background:**

Gastrointestinal stromal tumors (GISTs) account for less than 1% of all gastrointestinal tumors. The biological behaviors of GISTs vary from benign to malignant. GISTs are common in the stomach (55.6%) and small intestine (31.8%), but rarely in the rectum, colon (6%), and other sites (5.5%). Currently, the majority of published reports of primary appendiceal stromal tumors (PASTs) are case reports or case series.

**Methods:**

The PASTs described in this study were identified from a literature review (23 cases) and our center (one case). The relationship between PAST gross types and clinicopathological factors was analyzed and summarized. At the same time, the study also analyzed the related risk factors and survival of PASTs and GISTs.

**Results:**

Twenty-four cases of PASTs were compared with 254 cases of GISTs from our center. The results showed that there was a significant difference between the two groups in tumor size (*P* < 0.001), histological type (*P* = 0.013), CD34 expression (*P* < 0.001), and DOG-1 expression (*P* < 0.001). Disease-free survival (DFS) analysis of 11 cases of PASTs and 227 cases of GISTs found that a comparison of 3-year and 5-year DFS was not statistically significant (*P* = 0.894 and *P* = 0.846, respectively). In the DFS multivariate analysis, tumor mucosal ulceration, tumor size, and NIH risk classification were independent prognostic factors in 3-year and 5-year DFS.

**Conclusion:**

In this study, there was no significance in the survival of patients with appendix and gastric stromal tumors, which we hypothesized to be associated with the low sample size and incomplete follow-up records. Based on this, we conclude that the prognosis of primary appendiceal stromal tumors may be better than gastric tumors, but this needs to be confirmed in further prospective studies.

## Background

Gastrointestinal stromal tumors (GISTs) account for less than 1% of all gastrointestinal tumors and are generally considered to emanate from the interstitial cells of Cajal (ICCs) [1–3]. GISTs were first termed in 1983 by Mazur and Clark [4], who, using immunohistochemistry (IHC), discovered that the majority of gastric wall tumors are not derived from smooth muscle but instead are of nerve sheath origin. GISTs are classified into spindle cells (70%), epithelial cells (20%), and mixed cells (10%) by IHC and observation of histological characteristics under light microscopy [5].

The biological behaviors of GISTs vary from benign to malignant. CD117, CD34, and DOG1 expression is usually positive in IHC staining, and thus these proteins are useful when confirming diagnosis [2]. GISTs are common in the stomach (55.6%) and small intestine (31.8%), but rarely in the rectum, colon (6%), and other sites (5.5%) [6]. According to literature reports, PASTs are extremely rare [7] and without specific clinical symptoms. PASTs are often identified because of other diseases of the appendix (such as appendicitis or other tumors) or ileocecal tumor surgery [8]. Therefore, correct diagnoses of PASTs are very difficult to obtain prior to surgery. Currently, the majority of published reports of PASTs are case reports or case series. Therefore, this study aimed to assess the clinicopathological features and prognosis of PASTs.

## Materials and methods

The cases used in this study were identified through a review of databases and from our center. Cases were retrieved from Chinese and foreign databases. The Chinese databases were China National Knowledge Infrastructure (CNKI) (seven cases), VIP (eight cases), WANFANG DATA (13 cases), while the foreign databases included PubMed (12 cases) and EMBASE (four cases). After data synthesis, 20 reports were filtered [8–27], which included a total of 24 cases. One case of PAST that was identified during autopsy was excluded. From January 2009 to October 2017, our center reported only one case of PAST, a 59-year-old female patient, who received an exploratory laparotomy following the identification of a mass in the right lower quadrant upon CT examination for cervical cancer. During the exploration, a 10-cm-sized tumor was found on the appendix, with the ileocecal valve violated, and the patient received a right hemicolectomy and appendectomy. According to the National Institutes of Health (NIH) primary GIST standard [2, 28], this case was diagnosed as a high-risk appendiceal stromal tumor. Modified NIH risk classification is divided into categories according to tumor size and mitotic phase, as follows: very low risk, low risk, intermediate risk, and high risk [29].

The clinicopathological data of PASTs in this study included age, sex, tumor size, gross type, rupture, local ulceration, histological type, mitotic phase, NIH risk classification, gene mutation types, clinical symptoms, and survival data. For survival analysis, the exclusion criteria were as follows [30]: (1) stromal tumors with other sites, (2) the presence of other malignancies, (3) preoperative chemotherapy with imatinib, (4) no follow-up data, and (5) tumor rupture or metastasis before surgery. And inclusion criteria including (1) postoperative pathological diagnosis were PASTs and (2) R0 excision.

Statistical analysis was performed using SPSS 19.0 (SPSS Inc., USA). In this study, numerical variables were expressed as the mean ± SD. The *χ*^2^-test and Fisher exact test were applied to identify differences in clinicopathological parameters between GISTs and PASTs. Risk factors for survival were identified by univariate analysis and multivariate analysis using the Cox proportional hazards regression model. Estimations for disease-free survival (DFS: defined as the time from surgery to disease recurrence/death (months)) were obtained using the Kaplan-Meier method, and differences between Kaplan-Meier curves were investigated by log-rank test. *P* values of < 0.05 were considered to be statistically significant.

## Results

The clinicopathological features of the PASTs are shown in Table 1. A total of 24 cases of PASTs were included in this study. The patients’ age ranged from 7 to 88 years old (median, 59.17 years old) and tumor size ranged from 10 to 100 mm in maximum diameter; 11 cases exhibited tumors of less than 2 cm in diameter (45.8%) and only four cases (16.7%) were larger than 100 mm. Twenty tumors were solid (83.3%), and others were cystic (16.7%). Intraoperative exploration found that two cases of PASTs were ruptured, and appendix ulceration occurred in one case. The pathological results of the cases were spindle type (21/24, 87.5%), epithelial type (2/24, 8.3%), and mixed type (1/24, 4.2%). Only 17 of the 24 patients reported a mitotic index, with ≤ 5/50 HPF (high power field) in 14 cases (82.4%) and > 5/50 HPF in three cases (17.6%). Immunohistochemistry showed that 23 cases were CD117-positive (23/24, 95.8%), 15 were CD34-positive (15/20, 75%), and three were DOG-1-positive (3/5, 60%). There were only three cases with a mutation in exon 11 of gene encoding *KIT* and two wild-type mutations in all of the studies. According to the modified NIH risk classification and literature reports, 11 patients were at very low risk (45.8%), two patients were low-risk (8.3%), four patients were at intermediate risk (16.7%), and seven patients were at high risk (29.2%).

**Table 1:** Clinicopathological characteristics of 24 cases of PASTs

Characteristics	*N*(%)
Age (year)/(*Σ* = 24)	
<59	11(45.8)
≥ 59	13(54.2)
Sex (*Σ* = 24)	
Male	14(58.3)
Female	10(41.7)
Tumor size (cm)/(*Σ* = 24)	
≤ 2	11(45.8)
2.1–5.0	1(4.2)
5.1–10	8(33.3)
>10	4(16.7)
Gross type (*Σ* = 24)	
Solid	20(83.3)
Mixed	4(16.7)
Cystic	0(0.0)
Histologic type (*Σ* = 24)	
Spindle	21(87.5)
Epithelioid	2(8.3)
Mixed	1(4.2)
Lymph node metastasis (*Σ* = 24)	
Yes	1(4.2)
No	23(95.8)
Mitotic index(%)/(*Σ* = 17)	
≤ 5	14(82.4)
>5	3(17.6)
Ki-67(%)/(*Σ* = 6)	
<5	4(66.7)
≥ 5	2(33.3)
Immunohistochemistry (*Σ* = 24)	
CD117 (*Σ* = 24)	23(95.8)
CD34 (*Σ* = 20)	15(75.0)
DOG-1 (*Σ* = 5)	3(60.0)
SMA (*Σ* = 17)	4(23.5)
S-100 (*Σ* = 24)	7(29.2)
Mutational status (*Σ* = 5)	
Kit	3(60.0)
PDGFRA	0(0.0)
Wild type	2(40.0)
SDHB	0(0.0)
NIH risk category (*Σ* = 24)	
Very low risk	11(45.8)
Low risk	2(8.3)
Intermediate risk	4(16.7)
High risk	7(29.2)
Rupture (*Σ* = 24)	
Yes	2(8.3)
No	22(91.7)
Ulceration (*Σ* = 24)	
Yes	1(4.2)
No	23(95.8)
Symptoms (*Σ* = 24)	
Appendicitis	14(58.3)
Abdominal distension or pain or mass	17(70.8)
Hematochezia or anemia	3(12.5)
Nausea or emesis	3(12.5)
Others	6(25.0)

*PASTs* primary appendiceal stromal tumors, *NIH* National Institute of Health

The relationship between PAST gross types and clinicopathological factors were analyzed and are summarized in Table 2. According to the results of the analysis, there is no statistical significance (*P* > 0.05). We suspect that this may be related to the low sample size. The clinicopathological factors of 24 cases of PASTs such as age, sex, tumor size, histological type, mitotic index, CD117 expression, CD34 expression, DOG-1 expression, ulceration, and NIH risk classification were compared with 254 cases of GISTs from our center (Table 3). The results showed that there were significant differences between the two groups in tumor size (*P* < 0.001), histological type (*P* = 0.013), CD34 expression (*P* < 0.001), and DOG-1 expression (*P* < 0.001).

**Table 2:** The relationship between gross type and clinicopathologic characteristics of PASTs

Characteristics	Solid	Cystic	Mixed	*P*
Age (year)/(*Σ* = 24)				0.637
<59	9	0	2	
≥ 59	11	0	2	
Sex (*Σ* = 24)				0.094
Male	10	0	4	
Female	10	0	0	
Tumor size (cm)/(*Σ* = 24)				0.112
≤ 2	11	0	0	
2.1–5.0	1	0	0	
5.1–10	6	0	2	
>10	2	0	2	
Histologic type (*Σ* = 24)				0.064
Spindle	18	0	3	
Epithelioid	0	0	1	
Mixed	2	0	0	
Mitotic index (%)/(*Σ* = 24)				0.115
≤ 5	18	0	2	
>5	2	0	2	
Ki-67(%)/(*Σ* = 6)				0.445
<5	4	0	0	
≥ 5	2	0	0	
NIH risk category (*Σ* = 24)				0.089
VLR	11	0	0	
VL	2	0	0	
IR	2	0	2	
HR	5	0	2	
Ulceration (*Σ* = 24)				0.167
Yes	0	0	1	
No	20	0	3

**Table 3:** Comparison of clinicopathologic parameters between GISTs and PASTs

Characteristics	Appendix (*N* = 24)	Gastric (*N* = 254)	*P*
Age (year)			0.697
< 59	11.00	106	
≥ 59	13.00	148	
Sex			0.335
Male	14.00	122	
Female	10.00	132	
Tumor size (cm)			0.000
≤ 2	11.00	31	
2.1–5.0	1.00	90	
5.1–10	8.00	88	
> 10	4.00	43	
Censored	0.00	2	
Histologic type			0.013
Spindle	21.00	232	
Epithelioid	2.00	20	
Mixed	1.00	0	
Censored	0.00	2	
Mitotic index (%)			0.111
≤ 5	20.00	158	
> 5	4.00	89	
Censored	0.00	7	
CD117			0.556
+	23.00	226	
−	1.00	24	
Undetected	0.00	4	
CD34			
+	15.00	236	
−	4.00	12	0.000
Undetected	5.00	6	
DOG-1			0.000
+	3.00	182	
−	2.00	29	
Undetected	19.00	43	
Ulceration			0.008
Yes	1.00	74	
No	23.00	164	
Censored	0.00	16	
NIH risk category			0.000
VLR	11.00	24	
VL	2.00	66	
IR	4.00	75	
HR	7.00	88	
Censored	0.00	1	

**Fig. 1:**
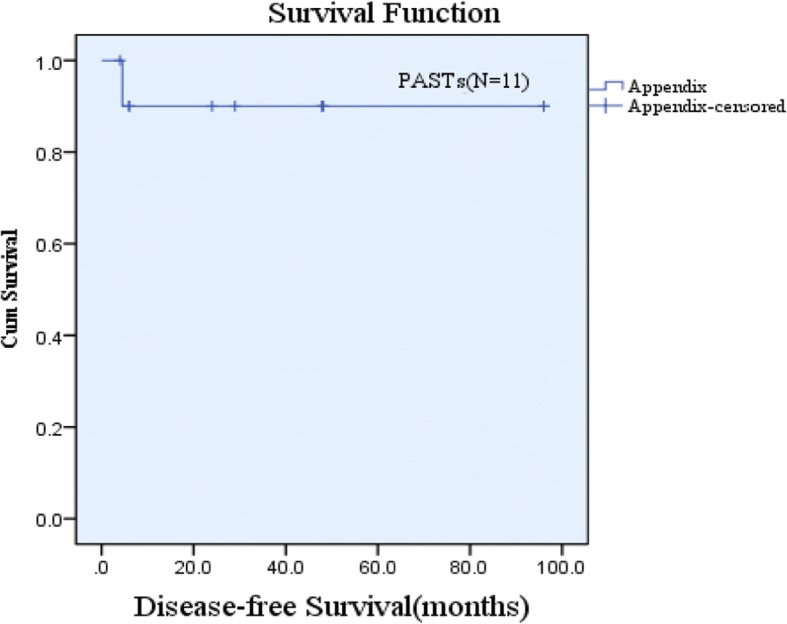
Disease-free survival (DFS) of primary appendix stromal tumors

**Fig. 2:**
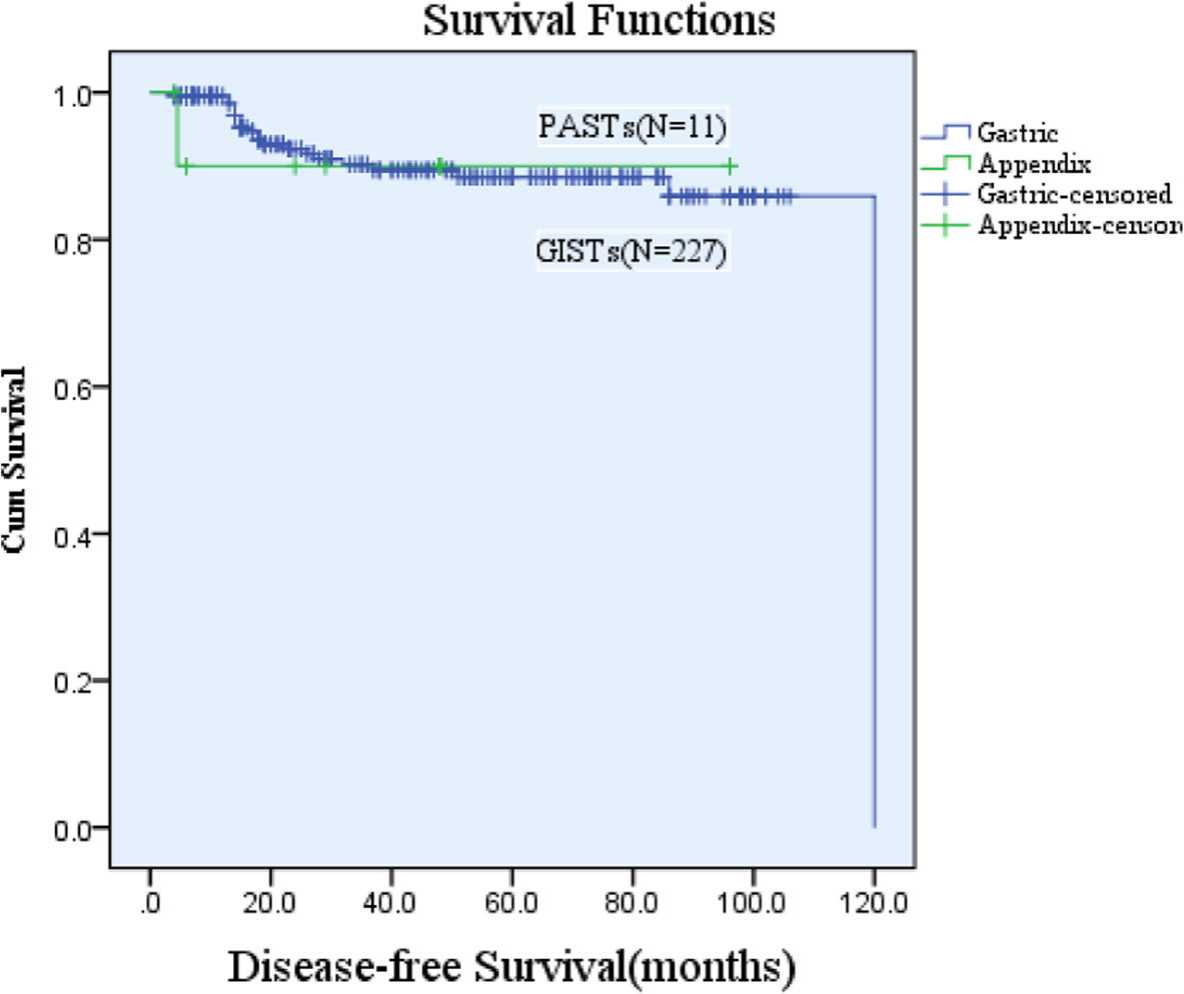
Disease-free survival (DFS) between primary appendix stromal tumors (PASTs) and gastric gastrointestinal stromal tumors (GISTs) (*P* = 0.846)

Finally, the survival data of 11 cases were selected for analysis according to the exclusion criteria. These patients had a DFS ranging from 4 to 96 months and a median DFS of 29 months (mean, 37.23 ± 34.10 months). The 3-year and 5-year DFS rates were 45.5% and 18.2%, respectively. The DFS of PAST patients was analyzed using Kaplan-Meier survival analysis and is shown in Fig. 1. Analysis of 11 cases of PASTs and 227 cases of GISTs found that the two groups of 3-year and 5-year DFS were not statistically significant (*P* = 0.894 and *P* = 0.846, respectively) (Fig. 2). In the DFS multivariate analysis (Table 4), tumor mucosal ulceration, tumor size, and NIH risk classification were independent prognostic factors in both groups.

**Table 4:** Univariate and multivariate analyses of prognostic factors for PASTs and GISTs

Prognostic factors	Univariate analysis			Multivariate analysis		
	*β*	HR(95%CI)	*P*	*β*	HR(95%CI)	*P*
DFS						
Age	0.633	1.883(0.730–4.860)	0.191			
Sex	−1.008	0.365(0.142–0.941)	0.037			
Location	0.192	1.210(0.162–9.030)	0.852			
Ulceration	−0.842	0.431(0.247–0.751)	0.003	−0.697	0.498(0.271–0.915)	0.025
Tumor size	−1.731	0.177(0.059–0.529)	0.002	1.054	2.868(1.166–7.054)	0.022
Mitotic index	1.569	4.802(1.952–11.814)	0.001			
Histologic type	1.249	3.488(1.380–8.816)	0.008			
NIH risk category	1.129	3.093(1.649–5.801)	0.000	0.955	2.598(1.402–4.815)	0.002

*DFS* disease-free survival

## Discussion

This study represented the largest number of PAST cases analyzed thus far. The clinicopathological features and prognosis of PASTs were statistically analyzed, and the survival rate of appendiceal stromal tumors was compared with that of gastric stromal tumors treated at our center; no difference was found between the two groups.

PASTs are extremely rare, constituting approximately 0.1% of all cancer diagnoses [7]. Other tumors also identified in the appendix include leiomyosarcoma, gastrointestinal stromal tumor, Kaposi’s sarcoma, granular cell tumor, gangliocytic paraganglioma, schwannoma, lipoma, hemangioma, and neural tumors. While PASTs are infrequently diagnosed, they cannot be neglected.

GISTs are generally considered to emanate from the interstitial cells of Cajal (ICC), which are pacemaker cells that regulate gut motility [31]. At present, there is no report about the origin of appendix stromal tumors. However, the appendix is part of the digestive tract, and thus gastrointestinal tumor data may have been combined with previously confirmed cases of appendix stromal tumors. We speculate that ICCs or ICC-like cells and multipotential mesenchymal stem cells also exist in the appendix. Of course, this conjecture requires further relative research to corroborate it.

PASTs usually present with nonspecific or appendicitis-like symptoms and lack of corresponding hematology detection marker. Therefore, correct diagnoses of PASTs are very difficult to obtain prior to surgery. In general, CT and magnetic resonance imaging (MRI) are the first choice to study tumor location and extension [32]. If the tumor is small, it is more difficult to find using CT or MRI, and because of their special anatomical structure, current endoscopy approaches are not yet suitable for this tumor type. The appendiceal small stromal tumors identified in our cases resulted from other diseases of the appendix (such as appendicitis or other tumors) or ileocecal tumor surgery. When tumor volume is large, it is not difficult to identify them using CT or MRI. Ultrasound or CT-guided fine needle aspiration (US/CT-FNA) may be helpful for the diagnosis of PASTs.

Immunohistochemical staining is useful to confirm the diagnosis of stromal tumors [33–35]. In GISTs, the positive rate of CD34 is about 50–80% and that of CD117 is 80–100% [36, 37]. The results of this study are similar, with 75% of cases CD34-positive and 95.8% CD117-positive. It has been shown that DOG-1 protein is characterized by high sensitivity (89%) and specificity (94.8%) relative to stromal tumor cell GISTs [33, 38], which is quite different to the results of our study, probably because of the low detection rate of DOG-1 (only five cases were tested). It was reported that *KIT* and *PDGFRA* gene mutations occurred in approximately 78.5% and 5–8% of GISTs, respectively [39]. In this study, there were only five cases of mutations (three cases of exon 11 mutations and two cases of wild type); thus, we did not study the gene mutation types further.

Complete surgical resection with negative microscopic margins is the standard treatment for GISTs [30, 40].Vassos et al [8] found that simple appendectomy was the standard treatment for most cases that were located in the body or tail of the appendix. In some cases, resection of adjacent tissue and organs or the base of the cecum may be necessary for complete removal of the tumor to minimize the risk of local recurrence. Chinese guidelines for the diagnosis and treatment of gastrointestinal stromal tumors indicate that lesions of less than 5 cm in diameter located in favorable anatomic sites, such as the greater curvature or anterior wall of gastric body and fundus, can be considered by laparoscopic method [41]. Considering the pathological features of cases in this study, 11 were small stromal tumors (45.8%) and more than half (54.2%) were located in the body or tail; thus, laparoscopic appendectomy may be feasible. However, relevant prospective clinical studies are needed to further confirm the feasibility and safety of laparoscopic surgery of PASTs. Since tumor rupture is an independent adverse prognostic factor [2, 28], surgery should follow the principle of “no touch, less compression.” Endoscopic application of an “extract bag” to avoid tumor rupture and spillage should be performed [41–43], and open surgery for resectable and over-sized stromal tumors is necessary.

It has been reported that tumor size, mitotic index, and tumor location are the best prognostic indicators for determining the malignant potential of GISTs [44], but the prognosis of appendix stromal tumors has not been described. The results of the multivariate analysis performed in this study showed that tumor ulcers, tumor size, and NIH grading were independent prognostic factors, and we compared the survival of appendix and gastric stromal tumors as well. However, since there are minimal overall survival (OS) data on appendix stromal tumors in these cases, we only performed a DFS analysis. There was no statistically significant difference in DFS between PASTs and GISTs. At present, because of the low numbers of appendix stromal tumor cases and incomplete follow-up records, the survival analysis of the present study may be different from the real clinical situation.

The current study has some limitations. This is a retrospective study with a short follow-up time, so the data integrity is limited. The sample size is not large enough, and some appendix stromal tumors are less than 1 cm in diameter, which will lead to sampling errors. Because the number of stromal tumor cases identified in other locations were limited at our center (particularly lower gastrointestinal stromal tumors), they could not be compared with appendix clinical pathology and survival characteristics.

## Conclusions

In this study, most of the PASTs were solid (20/24, 83.3%); there were no cystic cases, and most of the pathological diagnosis of PASTs were spindle cells (21/24, 87.5%). According to the NIH classification criteria, the median risk was more than 50% (13/24, 54.2%). By analyzing the data of PASTs and GISTs from our center, we found that there was a significant statistical difference between tumor size, histological type, CD34 expression, DOG-1 expression, ulceration, and NIH grade. Only one patient died of postoperative lymph node metastases in all selected cases. Rutkowski et al. [45] reported that the location of the primary tumor is an independent prognostic factor that affects the prognosis of GISTs. However, in this study, there was no significance in the survival of patients with appendix and gastric stromal tumors, which we hypothesized to be associated with the low sample size and incomplete follow-up records. Based on this, we conclude that the prognosis of primary appendiceal stromal tumors may be better than gastric tumors, but this needs to be confirmed in further prospective studies.

## References

[1] Sircar K, Hewlett BR, Huizinga JD, Chorneyko K, Berezin I, Riddell RH (1999). Interstitial cells of Cajal as precursors of gastrointestinal stromal tumors. Am J Surg Pathol.

[2] Lim KT, Tan KY (2017). Current research and treatment for gastrointestinal stromal tumors. World J Gastroenterol.

[3] Kindblom LG, Remotti HE, Aldenborg F, Meis-Kindblom JM (1998). Gastrointestinal pacemaker cell tumor (GIPACT): gastrointestinal stromal tumors show phenotypic characteristics of the interstitial cells of Cajal. Am J Pathol.

[4] Mazur MT, Clark HB (1983). Gastric stromal tumors. Reappraisal of histogenesis. Am J Surg Pathol.

[5] Corless CL, Fletcher JA, Heinrich MC (2004). Biology of gastrointestinal stromal tumors. Journal of Clinical Oncology. Proc Am Soc Clin Oncol.

[6] Søreide K, Sandvik OM, Søreide JA, Giljaca V, Jureckova A, Bulusu VR (2016). Global epidemiology of gastrointestinal stromal tumours (GIST): a systematic review of population-based cohort studies. Cancer Epidemiol.

[7] Misdraji J, Graemecook FM (2004). Miscellaneous conditions of the appendix. Semin Diagn Pathol.

[8] Vassos N, Agaimy A, Günther K, Hohenberger W, Schneider-Stock R, Croner RS (2013). A novel complex KIT mutation in a gastrointestinal stromal tumor of the vermiform appendix. Hum Pathol.

[9] Guo ALWZQ, Wang W (2001). A case of low grade appendix malignant stromal tumor. Chin J Pathol.

[10] Miettinen M, Sobin LH (2001). Gastrointestinal stromal tumors in the appendix: a clinicopathologic and immunohistochemical study of four cases. Am J Surg Pathol.

[11] He JFLSL (2002). A case of malignant tumor of appendix. Chin J Pathol.

[12] Wu YQWY (2004). A case of misdiagnosis of malignant stromal tumors in the ileocecal region. Chin J Misdiagnistics.

[13] Yap WM, Tan HW, Goh SG, Chuah KL (2005). Appendiceal gastrointestinal stromal tumor. Am J Surg Pathol.

[14] Guo HNLXM, Huang JYWJX (2006). One case of giant low grade gastrointestinal stromal tumor of appendix. J Diag PAthol.

[15] Kyu-Jong K, Park S, Park S, Hyun B, Kwon C (2007). Gastrointestinal stromal tumor of appendix incidentally diagnosed by appendiceal hemorrhage. World J Gastroenterol.

[16] Agaimy A, Pelz AF, Wieacker P, Roessner A, Wünsch PH, Schneider-Stock R (2008). Gastrointestinal stromal tumors of the vermiform appendix: clinicopathologic, immunohistochemical, and molecular study of 2 cases with literature review. Hum Pathol.

[17] Agaimy A, Wünsch PH, Dirnhofer S, Bihl MP, Terracciano LM, Tornillo L (2008). Microscopic gastrointestinal stromal tumors in esophageal and intestinal surgical resection specimens: a clinicopathologic, immunohistochemical, and molecular study of 19 lesions. Am J Surg Pathol.

[18] Elazary R, Schlager A, Khalaileh A, Appelbaum L, Bala M, Abu-Gazala M, Khatib A, Neuman T, Rivkind AI, Almogy G (2010). Malignant appendiceal GIST: case report and review of the literature. J Gastrointest Cancer.

[19] Yang F (2012). A case of Appendiceal stromal tumor. Chin Health Care Nutrition.

[20] Tran S, Dingeldein M, Mengshol SC, Kay S, Chin AC (2014). Incidental GIST after appendectomy in a pediatric patient: a first instance and review of pediatric patients with CD117 confirmed GISTs. Pediatr Surg Int.

[21] Back J, Jeanty J, Landas S (2015). Gastrointestinal stromal tumor of the appendix: case report and review of the literature. Human Pathology Case Reports.

[22] Luo WXZHH, Cui XHLDN (2015). Misdiagnosis of stromal tumors of gynecological tumors in 2 cases and literature review. Chin J Clin Obstet Gynecol.

[23] Pan YLXY. A case of low grade appendix malignant stromal tumor. Chin J Postgrad Med (z1). 2015:193–4.

[24] Zhu CY, Zhu YM (2015). Gastrointestinal stromal tumor of the vermiform appendix: a case report and literature review. World Chin J Digestol.

[25] Rahimi K, Gologan A, Haliotis T, Lamoureux E, Chetty R (2009). Gastrointestinal stromal tumor with autonomic nerve differentiation and coexistent mantle cell lymphoma involving the appendix. Int J Clin Exp Pathol.

[26] Chung JC, Song OP (2012). Gastrointestinal stromal tumor of the appendix. Turk J Gastroenterol.

[27] Bouassida M, Chtourou MF, Chalbi E, Chebbi F, Hamzaoui L, Sassi S (2013). Appendiceal GIST: report of an exceptional case and review of the literature. Pan Afr Med J.

[28] Fletcher CD, Berman JJ, Corless C, Gorstein F, Lasota J, Longley BJ, Miettinen M, O'Leary TJ, Remotti H, Rubin BP (2002). Diagnosis of gastrointestinal stromal tumors: a consensus approach. Hum Pathol.

[29] Joensuu H (2008). Risk stratification of patients diagnosed with gastrointestinal stromal tumor. Hum Pathol.

[30] Liu Z, Tian Y, Xu G, Liu S, Guo M, Lian X (2017). Pancreatic gastrointestinal stromal tumor: clinicopathologic features and prognosis. J Clin Gastroenterol.

[31] Miettinen M, Lasota J (2006). Gastrointestinal stromal tumors: pathology and prognosis at different sites. Semin Diagn Pathol.

[32] Demetri GD, Von MM, Antonescu CR, Dematteo RP, Ganjoo KN, Maki RG, Pisters PW, Raut CP, Riedel RF, Schuetze S (2010). NCCN task force report: update on the management of patients with gastrointestinal stromal tumors. J Natl Compr Canc Netw Jnccn 8 Suppl.

[33] Lopes LF, West RB, Bacchi LM, Van dRM, Bacchi CE (2010). DOG1 for the diagnosis of gastrointestinal stromal tumor (GIST): comparison between 2 different antibodies. Appl Immunohistochem Mol Morphol.

[34] Miettinen M, Lasota J (2006). Gastrointestinal stromal tumors: review on morphology, molecular pathology, prognosis, and differential diagnosis. Arch Pathol Lab Med.

[35] Rubin BP, Cooper K, Fletcher CD, Folpe AL, Gannon FH, Hunt JL (2010). Protocol for the examination of specimens from patients with tumors of soft tissue. Arch Pathol Lab Med.

[36] Hirota S, Isozaki K, Moriyama Y, Hashimoto K, Nishida T, Ishiguro S, Kawano K, Hanada M, Kurata A, Takeda M (1998). Gain-of-function mutations of c-kit in human gastrointestinal stromal tumors. Science.

[37] Sarlomo-Rikala M, Kovatich AJ, Barusevicius A, Miettinen M (1998). CD117: a sensitive marker for gastrointestinal stromal tumors that is more specific than CD34. Mod Pathol.

[38] Miettinen M, Wang ZF, Lasota J (2009). DOG1 antibody in the differential diagnosis of gastrointestinal stromal tumors: a study of 1840 cases. Am J Surg Pathol.

[39] Li K, Cheng H, Li Z, Pang Y, Jia X, Xie F (2017). Genetic progression in gastrointestinal stromal tumors: mechanisms and molecular interventions. Oncotarget.

[40] Valsangkar N, Sehdev A, Misra S, Zimmers TA, O'Neil BH, Koniaris LG (2015). Current management of gastrointestinal stromal tumors: surgery, current biomarkers, mutations, and therapy. Surgery.

[41] Jian Y, Jian W, Zhang S, Yingqiang Y, Xiaobo L (2017). Chinese consensus guidelines for diagnosis and management of gastrointestinal stromal tumor. Chin J Cancer Res.

[42] Ford SJ, Gronchi A (2016). Indications for surgery in advanced/metastatic GIST. Eur J Cancer.

[43] Huang CM, Chen QF, Lin JX, Lin M, Zheng CH, Li P, Xie JW, Wang JB, Lu J, Chen QY (2017). Can laparoscopic surgery be applied in gastric gastrointestinal stromal tumors located in unfavorable sites?: a study based on the NCCN guidelines. Medicine (Abingdon).

[44] Dematteo RP, Gold JS, Saran L, Gönen M, Liau KH, Maki RG, Singer S, Besmer P, Brennan MF, Antonescu CR (2008). Tumor mitotic rate, size, and location independently predict recurrence after resection of primary gastrointestinal stromal tumor (GIST). Cancer.

[45] Rutkowski P, Nowecki ZI, Michej W, Debiecrychter M, Woźniak A, Limon J, Siedlecki J, Grzesiakowska U, Kakol M, Osuch C (2007). Risk criteria and prognostic factors for predicting recurrences after resection of primary gastrointestinal stromal tumor. Ann Surg Oncol.

